# Rapid Diagnostics of Orthopaedic-Implant-Associated Infections Using Nanopore Shotgun Metagenomic Sequencing on Tissue Biopsies

**DOI:** 10.3390/microorganisms9010097

**Published:** 2021-01-04

**Authors:** J. Christopher Noone, Karin Helmersen, Truls Michael Leegaard, Inge Skråmm, Hege Vangstein Aamot

**Affiliations:** 1 Department of Microbiology and Infection Control, Akershus University Hospital, 1478 Lørenskog, Norway; Karin.Helmersen@ahus.no (K.H.); Truls.Michael.Leegaard@ahus.no (T.M.L.); Hege.Vangstein.Aamot@ahus.no (H.V.A.); 2 Faculty of Medicine, University of Oslo, 0316 Oslo, Norway; 3 Department of Clinical Molecular Biology (EpiGen), Akershus University Hospital and University of Oslo, 1478 Lørenskog, Norway; 4 Orthopaedic Clinic, Akershus University Hospital, 1478 Lørenskog, Norway; Inge.Skramm@ahus.no

**Keywords:** nanopore shotgun metagenomics, orthopaedic-implant-associated infections, rapid diagnostics

## Abstract

Conventional culture-based diagnostics of orthopaedic-implant-associated infections (OIAIs) are arduous. Hence, the aim of this study was to evaluate a culture-independent, rapid nanopore-based diagnostic protocol with regard to (a) pathogen identification, (b) time to pathogen identification, and (c) identification of antimicrobial resistance (AMR). This prospective proof-of-concept study included soft tissue biopsies from 32 patients with OIAIs undergoing first revision surgery at Akershus University Hospital, Norway. The biopsies were divided into two segments. Nanopore shotgun metagenomic sequencing and pathogen and antimicrobial resistance gene identification using the EPI2ME analysis platform (Oxford Nanopore Technologies) were performed on one segment. Conventional culture-based diagnostics were performed on the other. Microbial identification matched in 23/32 OIAI patients (72%). Sequencing detected additional microbes in 9/32 patients. Pathogens detected by culturing were identified by sequencing within a median of 1 h of sequencing start [range 1–18 h]. Phenotypic AMR was explained by the detection of resistance genes in 11/23 patients (48%). Diagnostics of OIAIs using shotgun metagenomics sequencing are possible within 24 h from biopsy using nanopore technology. Sequencing outperformed culturing with respect to speed and pathogen detection where pathogens were at sufficient concentration, whereas culture-based methods had an advantage at lower pathogen concentrations. Sequencing-based AMR detection may not yet be a suitable replacement for culture-based antibiotic susceptibility testing.

## 1. Introduction

Orthopaedic-implant-associated infections (OIAIs) are associated with a fivefold increased risk of 30-days to one-year mortality compared to aseptic implant failures [1]. Late and/or poorly targeted OIAI treatment can, in addition, lead to selection for antimicrobial-resistant bacterial strains, need of revision surgery, removal of the implant device, and loss of functionality [2,3].

Conventional culture-based diagnostic methods are comprehensive and often require several days to diagnose an OIAI. Detection is dependent on cultivating microbes, occasionally requiring growth conditions not easily reproduced in a laboratory setting. Culture-based methods are, therefore, impracticable for expedient routine diagnostics, extending the time to microbial identification and recommended targeted treatment [4,5]. A culture-independent diagnostic method could potentially reduce this time, thus expediting diagnosis and targeted treatment of OIAI patients.

There are a few culture-independent rapid diagnostic tools on the market. However, these are targeted approaches, limited to the detection of a pre-conceived set of microbes and antibiotic resistance genes [6,7,8]. Shotgun metagenomic sequencing is an untargeted method, free of these limitations. Here, all DNA in each sample is analysed, meaning that no a priori knowledge of the pathogen landscape is required. Shotgun metagenomic sequencing carried out with nanopore technology allows for the real-time analysis of sequencing data, which could facilitate same-day diagnostics of OIAIs.

Furthermore, conventional microbiological diagnostics of OIAIs involve the culturing of tissue biopsies. Tissue biopsies pose sequencing challenges as the human:pathogen DNA ratio is very high, even more so in OIAIs, where the use of implants can drastically reduce the number of bacteria needed to establish an infection [9,10]. We have previously published a DNA extraction protocol for use in sequencing directly from infected tissue, optimized for the removal of human DNA [11]. The protocol is tested herein on a larger cohort.

The aim of this study was to evaluate a culture-independent, rapid nanopore-based diagnostic protocol with regard to (a) pathogen identification, (b) time to pathogen identification, and (c) identification of antimicrobial resistance (AMR).

## 2. Materials and Methods

### 2.1. Patient Selection

In this prospective proof of concept study, all patients aged ≥18 years undergoing first revision surgery for acute OIAIs (including prosthetic joint infections, fracture implants, and osteotomy implants) from January 2017 to December 2018 at Akershus University Hospital (Ahus), Norway, were eligible for inclusion. Ahus is Norway’s largest acute care hospital and performs approximately 4000 orthopaedic implant surgeries annually. Diagnostic and clinical data were collected retrospectively from the hospital’s diagnostic and clinical databases. These patients were selected according to the criteria for OIAIs as described by Parvizi [12]. Cultivation results have previously been reported [5]. In addition, biopsies from 15 of the patients have been analysed on a rapid molecular diagnostics platform [8]. Three patients’ biopsy shotgun metagenomic sequencing results have been previously published (Supplementary Materials, Table S1, IDs 117, 127, 130) [11].

Tissue biopsies from five patients undergoing total hip arthroplasty with no signs of infection were included as negative controls.

**Figure 1:**
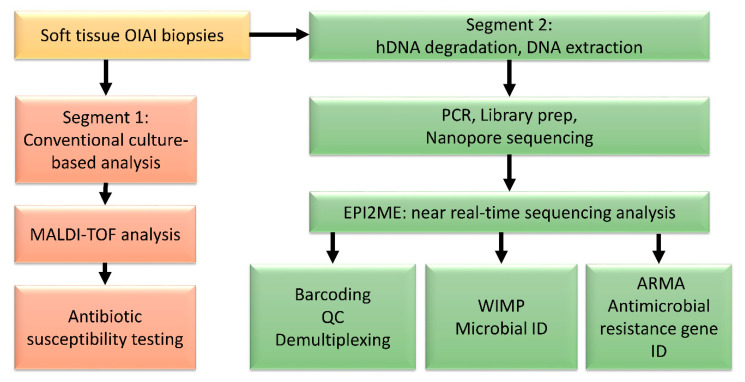
Flow chart of the parallel workflows carried out on the biopsies from orthopaedic-implant-associated infections (OIAI). Segment 1 followed conventional microbiological diagnostics, and segment 2 the nanopore shotgun metagenomic protocol. Abbreviations are as follows: MALDI-TOF (matrix-assisted laser desorption ionization-time of flight), hDNA (human DNA), QC (quality control), WIMP (What’s in My Pot; taxonomical identification), ARMA (Antimicrobial Resistance Mapping Application).

As part of conventional diagnostics, up to five soft tissue biopsies were taken from each patient. Additional material such as synovial fluid and bone biopsies may also be part of conventional diagnostics, however, only tissue biopsies were included in this study for comparison purposes. The biopsies were taken from areas directly adjacent to the infected implant and further divided in two. One segment was analysed following conventional microbiological diagnostics, the other, earmarked for sequencing, was initially frozen at −80 °C (Figure 1).

Conventional culturing was performed by homogenizing the tissue samples individually with mortar and pestle in heart infusion broth (HIB), done in a type 2 microbiological safety cabinet with subsequent seeding and matrix-assisted laser desorption ionization time of flight (MALDI-TOF) analysis. Incubation was terminated after 5 days following consensus [12] except in cases where slow growing bacteria, such as *Cutibacterium acnes*, were deemed clinically relevant. The incubation period was prolonged to 14 days in such cases. Semi-quantitative assessments of culture growth were performed and were described in ascending order of growth density as single-spread colonies, sparse, moderate, and rich.

Antibiotic susceptibility testing (AST) was performed in accordance with guidelines from the European Committee on Antimicrobial Susceptibility Testing [13]. Breakpoints and definitions were used to categorize the isolate as sensitive (S), intermediate (I), or resistant (R). AMR phenotype and genotype comparisons were performed only in patients where AST was carried out on tissue biopsies.

### 2.2. DNA Extraction

DNA extraction was carried out using the Ultra-Deep Microbiome Prep kit (Molzym, Bremen, Germany). The extraction process was performed in a laminar airflow cabinet, following the manufacturer’s protocol with optimization as described previously [11].

DNA was extracted from a single patient’s biopsies in each session to avoid cross-contamination. All but seven extraction sessions included an extraction negative, consisting of a tissue-free tube containing all the reagents of the extraction process, subjected to the same conditions as the patient samples.

### 2.3. Nanopore Sequencing and Analysis

Library preparation was carried out using the SQK-RPB004 Rapid PCR Barcoding Kit (ONT, Oxford, UK). The input volume was 3 µL of undiluted isolated nucleic acids. DNA concentrations were measured with Qubit 3 (Thermo Fisher Scientific, Waltham, MA, USA). The manufacturer’s protocol (RPB_9059_v1_revG_08Mar2018) was strictly adhered to except that the pre-PCR dilution of the isolated nucleic acids was omitted in order to carry as much bacterial DNA as possible downstream to sequencing.

All biopsies from a single patient, an extraction negative (where applicable), and a no template control (NTC) were sequenced on a single flow cell. Shotgun metagenomic sequencing was performed using R9.4.1 FLO-MIN 106 flow cells on either the MinION or GridION platform (ONT, Oxford, UK). Each sequencing run lasted for 48 h.

MinKNOW software with Guppy basecaller was used for sequencing data analysis. See Supplementary Materials for software versions (Table S2). Workflows in the cloud-based bioinformatics platform EPI2ME were used for demultiplexing (Barcoding), identification of pathogen (WIMP, What’s in My Pot), and AMR genes (ARMA, Antimicrobial Resistance Mapping Application). The three EPI2ME workflows were employed using a default Q-score ≥7. ARMA results were set at ≥90% average alignment accuracy, based on the findings of Schmidt and co-workers [14]. The protein homolog model was employed.

Extraction negative controls, NTCs, and negative patient samples were assessed to identify background microbial DNA contaminants from the environment and kit reagents. All microbial DNA not detected in negative controls were considered to be from potential pathogens. Drawing on our lab’s experience with the routine diagnostic metagenomic 16S sequencing assay direct on patient sample material, species common to both controls and biopsies were discounted if not present in quantities greater than 10-times the quantity of the negative control in two or more of a patient’s biopsies.

A patient’s sequencing results were classified positive when a minimum of two biopsies were positive for identical species.

The protocol was first tested in a pilot study using biopsies from six random patients with culture-positive biopsy results, all four patients with culture-negative biopsies results, and the biopsies from five infection-free patients described earlier.

## 3. Results

Of a possible 84 patients, 155 biopsies from 32 patients were included. Results of all cultivation and shotgun metagenomic sequencing are detailed in the Supplementary Materials (Table S1). Negative sequencing results are described herein in terms dismissive of the putative background DNA signal that will be described in further detail below. See Supplementary Materials for nanopore sequencing QC metrics (Table S3).

Where detectable, the extracted DNA concentration ranged from 0.045 to 48 ng/µL (median 0.27 ng/µL). The eluate from 57 biopsies was below the instrument’s limit of detection.

### 3.1. Pilot Study

Due to the low number of patients with culture-negative biopsies, all were included. Conventional culturing and shotgun metagenomic sequencing were in agreement in six patients (IDs 101, 109, 116, 118, 121, 137). Biopsies from three patients (IDs 107, 120, 124) were sequencing positive for the cultured pathogen in one or fewer biopsies. In the culture-negative biopsies from one patient (ID 142), sequencing was able to detect a putative pathogen. All biopsies from the negative control patients (IDs 200–204) were culture and sequencing negative. AMR gene identification agreed between methods in four patients, partially in three patients, and not at all in three patients. Ambiguity regarding the protocol’s feasibility as a rapid replacement for conventional methods and background noise in the data made interpretation of a small dataset difficult, so the pilot study was merged with the remainder of the 32 OIAI patients.

### 3.2. Microbial Identification

**Figure 2:**
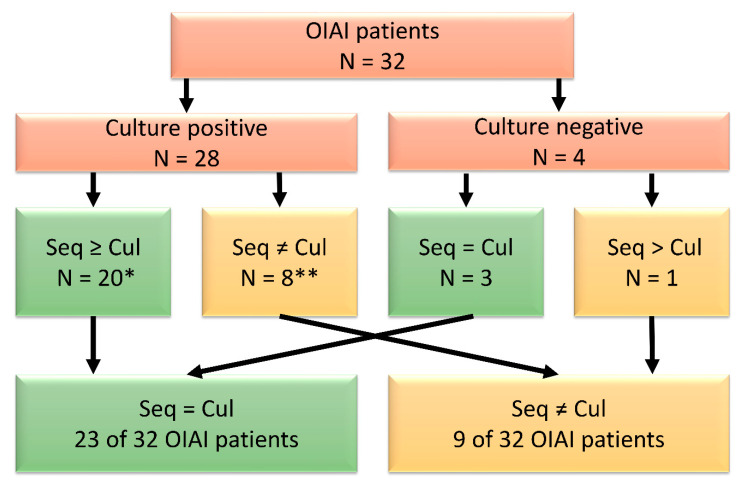
Comparison of microbial identification results of shotgun metagenomic sequencing (Seq) and culturing (Cul) from all 32 patients with orthopaedic implant-associated infections (OIAI). A positive result requires that identical pathogens are detected in a minimum of 2 biopsies from the same patient. Seq > Cul indicates that sequencing resulted in the detection of additional putative pathogens. * Includes Seq > Cul (N = 8); ** Seq ≠ Cul includes patients where sequencing detected 1 of 2 cultured species (N = 2), detected the cultured species in 1 biopsy (N = 2), and failed to detect the cultured species (N = 4).

A graphic comparison of microbial identification results between shotgun metagenomic sequencing and conventional culturing is depicted in Figure 2.

Overall, in 23 of the 32 patients (72%), nanopore sequencing detected either the same putative pathogens as conventional diagnostics or confirmed the samples as negative. Sequencing detected additional putative pathogens in nine of 32 patients (28%). Included in this number is the specification of the individual species collectively identified as group-B streptococci by one patient’s (ID 135) conventional diagnostics. Shotgun metagenomic sequencing identified *Staphylococcus aureus* as the most common pathogen (50% of patients) followed by *Staphylococcus epidermidis* and *Staphylococcus lugdunensis* (9% of patients each).

Three patients’ (IDs 104, 114, 140) biopsy sequencing results revealed the presence of *S. epidermidis* in addition to the single microbe detected by conventional methods. Other instances of previously undetected species in poly-staphylococci OIAIs revealed by shotgun metagenomic sequencing included three *S. lugdunensis* (IDs 114, 140, 141), and two *Staphylococcus argenteus* (IDs 114, 140), in addition to the staphylococci detected by culturing. Patient 140′s sequencing results also revealed the relatively low-grade presence of several other staphylococci (Supplementary Materials, Table S1). Two patients’ sequencing results indicated, what appear to be unrelated concurrent infections. Their sequencing results revealed additional microbes, dominated by Gram-positive anaerobic cocci and various fungal species, over 2000 distinct microbes in all (IDs 121, 135). The fungi in patient 135 were localized to one biopsy (V2), however.

Conventional diagnostics were able to detect bacterial species in eight of the 32 OIAI patients, whose sequencing results did not meet the criterion for positive. Of these, two patients’ sequencing results were positive for the conventionally detected bacteria in only one biopsy (IDs 107, 134). In both cases, the species in question was not present in the negative controls, nor was it a member of the putative background DNA. Two patients’ sequencing results confirmed one of two cultured microbes. One (ID 123) was positive for both *S. aureus* and β-haemolytic streptococci using conventional methods. Nanopore results detected only *S. aureus*. In the other (ID 125), sequencing again detected the cultured *S. aureus*, but not the *Micrococcus luteus* cultured from enrichment broth. The remaining four pathogens escaped detection.

A variety of putative background or contaminant DNA was observed across virtually all OIAI patients and infection-free patient controls, as well as many extraction negative controls and NTCs. This background microbial DNA most frequently included varying amounts of (percent of patients positive for each) *Escherichia coli* (95%), *Malassezia globosa* (95%), *Arthrobacter* spp. (89%), *Cutibacterium acnes* (86%), *Bacillus subtilis* (78%), *Moraxella osloensis* (76%), *Mucilaginibacter* spp. (73%), *Klebsiella* spp. (65%), *Bacillus cereus* (30%), and *Corynebacterium* spp. (27%).

### 3.3. Time to Results of Sequencing

In terms of culture growth, cultured species described here as “rich” were consistently detected within an hour of sequencing start. Sequencing start to detection of cultured pathogens took a median time of one hour [range 1–18 h]. Together with the six hours of pre-sequencing DNA extraction and library preparation, this amounts to a maximum total time of 24 h from biopsy to result.

Sequencing detected species undetected by conventional diagnostics in nine patients’ biopsies. These additional microbes were detected by nanopore sequencing after a median of five hours [range 1–14 h] from sequencing start. In 16 of the 20 patients where sequencing results agreed with conventional methods, nanopore results would have been available within two hours of sequencing start.

### 3.4. Antimicrobial Resistance

AMR detection results are detailed in the Supplementary Materials (Table S4). Of the 32 OIAI patients, four matched in that neither AST nor sequencing detected any resistance (IDs 110, 133, 134, 136). No AST could be carried out on the four patients whose biopsies were culture-negative (IDs 118, 137, 142, 101). One patient’s AST was inconclusive (ID 115). The remaining 23 patients’ biopsies resulted in either an antimicrobial resistant phenotype or genotype. Of these, 11 resistance phenotypes could be partially explained by the respective bacterial genotypes. Genotypes that explained observed phenotypic resistance to penicillin, tetracycline, and ciprofloxacin were detected. A complete discrepancy between phenotype and genotype was observed in pathogens in 11 patients. Four of the 11 discrepancies were the result of sequencing negative patient samples (IDs 105, 107, 120, 124). The remaining seven discrepancies were from low culture growth densities: single-spread colonies (IDs 109, 121, 123, 130) and sparse growth (IDs 104, 112, 117). A putative explanatory genotype for one patient’s samples’ AST results was detected slightly under the 90% identity cut-off (ID 135).

Ten patients’ biopsy sequencing results were accompanied by the detection of AMR genes that were not reflected in phenotypic testing (IDs 111, 112, 114, 116, 122, 125, 130, 139, 140, 141): *tet38*, *mgrA*, *arlS*, *arlR*, *mepA*, and *mepR*.

AMR genes *tetC*, *bla*_TEM-4_, and *APH(3′)-Ia(3)* were also evident in the putative background DNA. These AMR genes were detected in the sequencing results of 86% (*tetC*), 92% (*bla*_TEM-4_), and 57% (*APH(3′)-Ia(3)*) of patients and controls.

## 4. Discussion

This proof-of-concept study demonstrates that rapid diagnostics of OIAIs using nanopore shotgun metagenomic sequencing on tissue biopsies are possible. Overall, in 72% of OIAI patients’ biopsies, nanopore sequencing results were in agreement with conventional culturing. Additional microbes were detected in nine patients’ biopsies, including one patient with culture-negative biopsies. Conventionally detected pathogens were detected by nanopore sequencing in a median of one hour [range 1–18 h], and additional microbes after a median of five hours [range 1–14 h] from sequencing start. AMR phenotype was plausibly explained by genotype in 11 of the 23 AMR-positive patients.

The threshold for nanopore detection of OIAI pathogens seems to lie between pathogen concentrations requiring pre-cultivation in enrichment broth for conventional detection and those resulting in what is described as “single-spread colonies”. Patient 120 was the only instance where a cultured microbe described as “sparse growth” went undetected by sequencing. Conversely, cultures described as “single-spread colonies” and those requiring broth enrichment were detected in as little as one hour from sequencing start in some instances (IDs 104, 130, 135). All species that went undetected by shotgun metagenomic sequencing were Gram-positive, possibly indicating a slight bias in DNA extraction only relevant at very low biopsy pathogen concentrations.

The chosen cut-off of a required 10-times greater read count of bacteria common to both a patient biopsy and the respective sequencing run’s negative controls seems appropriate. An exception was found, however, in one patient (ID 116), whose respective cultures were positive for *S. aureus*. *S. epidermidis* was among the additional bacteria detected by sequencing. The run’s extraction negative control contained one *S. epidermidis* read. *S. epidermidis* was present at a 10-times greater read count in only one biopsy. *S. epidermidis* is not a member of the putative background, but its below cut-off presence in the patient’s biopsies prevented it from being counted as an additional species detected.

Contaminant DNA has been reported both to be ubiquitous in commonly used reagents and also to vary both between different kits and different lots of the same kit [15]. We found a background signal in virtually all samples. *Malassezia* *globosa*, for example is a commonly encountered member of the human microbiota. The presence of *E. coli*, *C. acnes,* and *K. pneumoniae* background is potentially confounding. All three are both opportunistic pathogens as well as commonly encountered sources of environmental and PCR/sequencing contamination [16,17,18,19]. The polymerase used in our protocol’s PCR lists *E. coli* as the source of the enzyme’s expression under production, thus contributing artefact DNA as a consequence [20]. The fact that these frequently detected organisms completely escaped detection by culturing is further reason to interpret them as contamination. Among other sequences interpreted as contaminant DNA were sequences from *B. subtilis*, *Arthrobacter* spp., *Corynebacterium striatum*, *Mucilaginibacter* spp., *B. cereus*, and *Moraxella. osloensis*, all of which are documented reagent and environmental contaminants [15,21,22,23]. A complete list of the microbial DNA interpreted as background is included in the Supplementary Materials (Table S1).

In 16 of the 20 patients where sequencing results agreed with conventional methods, nanopore bacterial identification results would have been available within two hours of sequencing start. A median time to pathogen identification of one hour from sequencing start and a maximum time of 18 h, amounts to a total time to pathogen identification of 7 to 24 h when added to the six hours of pre-sequencing DNA extraction and library preparation.

In general, 24 h to pathogen identification is well within the parameters of rapid diagnostics. In addition, it is a significant reduction from the 5-day culturing required for a negative result under conventional methods. Negative biopsy results could arguably be reported after 24 h.

None of the three AMR models included in EPI2ME were able to unambiguously predict the phenotypic results of conventional testing. Instances of discord between phenotypic and genotypic resistance have been observed throughout this study, as well as in the work of others [14,24]. A more careful analysis of the variation present within the detected genes might have yielded a more accurate prediction of these gene’s ability to confer resistance. However, this is an assessment of shotgun metagenomic sequencing and the EPI2ME data analysis workflow as a rapid replacement protocol for conventional OIAI diagnosis as it stands. The 90% AMR gene identification cut-off appears approximately appropriate. However, AST in one patient’s samples (ID 135) revealed resistance to erythromycin and clindamycin, and it seems the 90% cut-off was slightly too stringent for the detected corresponding *lmrP* gene (88.8% match) [25].

Several results were accompanied by the detection of AMR genes that were not reflected in phenotypic testing (IDs 111, 112, 114, 116, 122, 125, 130, 139, 140, 141). Genes such as *tet38*, *mgrA*, *arls*, *arlR*, *mepA*, and *mepR* are reported to be present in in excess of 99% of the *S. aureus* genomes by the Comprehensive Antibiotic Resistance Database (https://card.mcmaster.ca), so we would expect to have found them in virtually all the *S. aureus* genomes detected here had the OIAI bacterial load been sufficient.

There are several possible reasons behind the discrepancy between AMR genotype and phenotype. Input DNA concentration was well below the recommended 1–5 ng. This may well have resulted in too few reads from a given pathogen, leading to its AMR genotype escaping detection. It stands to reason that where sequencing failed to detect the cultured pathogen, it would also fail to detect the organism’s AMR gene(s). Four of the 11 discrepancies were the result of sequencing negative patient samples (IDs 105, 107, 120, 124). In the sequencing positive samples where no AMR genes were detected to explain phenotypic AMR (IDs 104, 109, 112, 117, 121, 123, 130), the pathogens were at the putative culture growth threshold for sequencing detection. The discrepancy is likely due to insufficient breadth of sequencing coverage.

Despite the hDNA degradation steps of the extraction protocol, the majority of reads were human. Further optimization of hDNA removal could conceivably lead to a lower hDNA to bacterial DNA ratio and improved microbial DNA amplification during PCR, thus permitting better microbial genotyping. Additionally, lower hDNA to bacterial DNA ratios could foreseeably allow for more rapid bacterial identification, allowing bacterial DNA proportionally increased access to the sequencing pores.

An additional consideration is that the freeze–thaw cycle in the protocol has the potential to lyse both human and bacterial cells. Extracellular DNA is degraded in the first steps of the DNA extraction protocol. This can have removed some of the already sparse bacterial DNA, further contributing to the suboptimal bacterial DNA concentration in some samples. Analysis of fresh tissue biopsies would be expected to result in both additional hDNA reads as well as additional bacterial DNA reads in the absence of the freeze–thaw cycle. The protocol should thus be tested, comparing fresh and frozen tissue biopsies to determine the extent of this effect.

It is also plausible that factors independent of genotype influence the detection of phenotypic AMR. Pharmacokinetic and pharmacodynamic parameters and the absence of selective pressures in a culture can influence the observed phenotype [26,27]. Additionally, conventional AST was performed on a single biopsy from each patient. Hence, biopsy selection and, consequently, the resulting cultures may not fully represent the diversity of bacterial sub-species present in an OIAI.

Accepted cut-off levels of gene identification via sequence similarity alone are clearly inadequate to fully predict phenotypic AMR. A single variation in a gene is sufficient to either confer or disable a bacterium’s antimicrobial properties [28,29]. Thus, our choice of a 90% cut-off for the identification of AMR genes could have contributed to the discrepancy between AMR genotype and phenotype, as in the case of patient 135.

The putative DNA background was also evident in the results of AMR gene analysis. Three resistance genes were repeatedly detected in sequencing results: *bla*_TEM-4_, *tetC*, and *APH(3′)-Ia(3)*. The three genes were present across culture-positive and culture-negative biopsies, extraction negatives, and NTCs. All three are common to Gram-negative species, namely the *E. coli* and *Klebsiella* spp. virtually ubiquitous to the background DNA [30,31,32], further emphasizing the importance of analysing infection-free patient samples and the inclusion of negative controls.

Few other studies have investigated the use of nanopore shotgun metagenomic sequencing as a diagnostic tool for OIAIs and then on sample types other than tissue biopsies. Street and co-workers, using Illumina’s MiSeq platform, tested metagenomic sequencing on sonication fluid in the diagnostics of prosthetic joint infections [33]. They also noted the confounding effects of contamination DNA from commensal/environmental/reagent sources. As in our work, Street and co-workers were able to detect several bacterial species that went undetected by culturing, notably several anaerobic species, suggesting also that in certain contexts metagenomic sequencing may be more sensitive than culturing. They were unable to identify an organism cultured from sonication fluid in eight of 97 instances, and results were in complete agreement in 68% of the samples, similar to our study. Nine of the sonication fluid samples from this study were further analysed with nanopore shotgun metagenomic sequencing. Although a different analysis pipeline was implemented, results indicated a speed of pathogen identification comparable to that of our study [34].

While the compartmentalized cost difference between nanopore metagenomic sequencing and culture-based diagnostics is meaningful, we contend that a more holistic cost analysis is warranted. The associated additional costs of delayed or inappropriate OIAI treatment must be taken into account. More rapid diagnosis and administration of targeted treatment lead potentially to shortened duration of treatment, reduced functional impairment, and shorter hospital stays, all of which translate to reduced costs to a healthcare system. Future studies are to test an alternate sample material and ONT’s Flongle flow cell insert system, which can potentially drive costs down significantly.

This study is a single-centre study and carried out in a country with a low prevalence of AMR bacteria. A greater prevalence of AMR genes might have given better insight into the ARMA workflow’s ability to predict resistance phenotype. Inclusion of only 32 of 84 eligible patients has the potential to cause an unintentional bias, but the cultivation results of the previously presented larger patient group [5] suggest that our subgroup is representative of OIAIs from this geographic area.

## 5. Conclusions

Diagnostics of OIAIs using shotgun metagenomics sequencing are possible within 24 h from biopsy using nanopore technology. The sequencing workflow tested here outperformed culturing in all instances with respect to additional microbes detected and the speed at which they were detected when pathogens were at sufficient concentration. Culture-based methods had an advantage only when pathogens were at lower concentrations. Sequencing-based AMR detection may not yet be a stand-alone replacement for culture-based AST.

## Data Availability

All relevant data are included in the paper and the Supplementary Materials. Due to the presence of human sequences from patients, sequencing data cannot be publicly shared. Further inquiries can be addressed to the corresponding author.

## Supplementary Materials

The following are available online at https://www.mdpi.com/2076-2607/9/1/97/s1: Table S1: Results of microbial identification across conventional microbiological methods and nanopore shotgun metagenomic sequencing for all OIAI patients’ biopsies; Table S2: Bioinformatic program versions used; Table S3. Nanopore sequencing QC metrics; Table S4. Overview of all patients whose biopsies were positive for either an antimicrobial resistance (AMR) phenotype from conventional antibiotic susceptibility testing (AST) or AMR genotype from shotgun metagenomic sequencing.
