# Supplementary material for: Rapid Diagnostics of Orthopaedic-Implant-Associated Infections Using Nanopore Shotgun Metagenomic Sequencing on Tissue Biopsies
Source: Microorganisms. 2021 Jan 4;9(1):97. doi: 10.3390/microorganisms9010097 (PMC7823515; doi:10.3390/microorganisms9010097)
Supplement: Supplementary file 1 [file microorganisms-09-00097-s001.pdf]

Supplemental data

Rapid diagnostics of orthopaedic implant-associated infections using nanopore shotgun metagenomics sequencing on tissue biopsies

J Christopher Noone[1,2]*, Karin Helmersen[1,3], Truls Michael Leegaard[1,2], Inge Skråmm[4], Hege Vangstein Aamot[1]

[1]Department of Microbiology and Infection Control, Akershus University Hospital, Lørenskog, Norway

[2]Faculty of Medicine, University of Oslo, Oslo, Norway

[3]Department of Clinical Molecular Biology (EpiGen), Akershus University Hospital and University of Oslo, Lørenskog, Norway

[4]Orthopaedic clinic, Akershus University Hospital, Lørenskog, Norway

Corresponding author

J. Christopher Noone

Department of Microbiology and Infection Control

Akershus University Hospital

1478 Lørenskog, Norway

Email: cnoo@ahus.no

Telephone +47 924 80 857

Table S1. Results of microbial identification across conventional microbiological methods and nanopore shotgun metagenomic sequencing for all OIAI patients' biopsies. Culture growth densities are termed as follows: rich, moderate (mod), sparse, spread single colonies (SSC), and requiring pre-cultivation in enrichment broth (broth). V* indicates the name of the individual tissue biopsy, NA (not applicable), NTC (no template control), and EN (extraction negative control).

**Patient ID**	**Biopsy/control ID**	**Nanopore results: putative pathogens, reads**	**Culture results, growth**	**Nanopore results: putative background (species, reads)**	**Nanopore results: human DNA reads**
**101**	VB	Negative	Culture negative	*E. coli*, 4; *Arthrobacter sp.*, 2; *Mucilaginibacter sp.*, 1	6
**101**	V2	Negative	Culture negative	*E. coli*, 15; *Arthrobacter sp.*, 1;	3,699

				Mucilaginibacter sp., 1	
**101**	V3	Negative	Culture negative	*E. coli*, 2; *Arthrobacter sp.*, 1	161
**101**	V4	Negative	Culture negative	*E. coli*, 5	46
**101**	V5	Negative	Culture negative	*E. coli*, 12	60
**101**	EN	Negative	NA	*E. coli*, 3; *Arthrobacter sp.*, 1	18
**101**	NTC	Negative	NA	*E. coli*, 5; *Arthrobacter sp.*, 2; *Mucilaginibacter sp.*, 1	2
**104**	VB	*S. lugdunensis,* 44; *S. epidermidis,* 6; *C. acnes*, 2; others <2	*S. Lugdunensis,* sparse	*E. coli*, 8; others <2	89
**104**	V2	Negative	*S. Lugdunensis,* sparse	Negative	
**104**	V3	*S. lugdunensis,* 306; *S. epidermidis,* 5; *C. acnes*, 1	*S. Lugdunensis,* sparse	*E. coli*, 10; others, 1	308
**104**	V4	*S. lugdunensis,* 24;	*S. Lugdunensis*, ssc;	*E. coli*, 5; others, <2	168

		C. acnes, 2	*C. acnes*, ssc		
**104**	V5	*S. lugdunensis,* 58;<br>*S. epidermidis,* 2	*S. Lugdunensis,* sparse	*M. globosa*, 160; *E. coli*, 2;<br>others, <2	46283
**104**	V6	Negative	*S. Lugdunensis,* sparse;<br>*C. acnes* ssc	*E. coli*, 6; others, 1	36
**104**	NTC	Negative	NA	Negative	
**105**	VB	Negative	Culture negative	*E. coli,* 4	821
**105**	V2	NA	*S. Aureus,* broth;<br>*S. epidermidis,* broth	NA	
**105**	V3	NA	*S. Aureus,* broth;<br>*S. epidermidis,* broth	NA	
**105**	V4	*S. aureus,* 49	*S. Aureus,* broth;<br>*S. epidermidis,* broth	*E. coli,* 24	67150
**105**	V5	Negative	*S. Aureus,* ssc;<br>*S. epidermidis,* broth	*E. Coli*, 16	10235
**105**	EN	Negative	NA	*E. coli,* 8;	1

				Mycobacterium abscessus,2	
**105**	NTC	Negative	NA	*E. coli*,1;	1
**107**	VB	*S. epidermidis*, 33;<br>*S. aureus*, 2	*S. epidermidis*, ssc	*E. coli*, 32; *B. subtilis*, 5;<br>*Arthrobacter sp.*, 5;<br>*M. globosa*, 4;<br>*Mucilaginibacter sp.*, 2	1944
**107**	V2	Negative	*S. epidermidis*, broth	*E.coli*, 31; *Arthrobacter sp.*, 2;<br>*Bacillus mycoides*, 2	479
**107**	V3	Negative	*S. epidermidis*, ssc	*E.coli*, 31; *Arthrobacter sp.*, 3;<br>*B. subtilis*, 3;<br>*Mucilaginibacter sp.*, 2	1173
**107**	V4	Negative	*S. epidermidis*, broth	*E. coli*, 16; *Arthrobacter sp.*, 3;<br>*Mucilaginibacter sp.*, 3	15
**107**	V5	Negative	Culture negative	*E coli*, 97; *B. subtilis*, 4;<br>*Mucilaginibacter sp.*, 4;<br>*M. globosa*, 2; *Arthrobacter sp.*, 4;	97

				Bacillus mycoides, 2	
**107**	EN	Negative	NA	*E coli*, 15; *Arthrobacter sp.*, 2; *C. acnes*, 2; *B. mycoides*, 2;	4
**107**	NTC	Negative	NA	*E coli*, 13; *B. subtilis*, 4; *Mucilaginibacter sp.*, 2; *Arthrobacter sp.*, 2; *Bacillus mycoides*, 1	
**108**	VB	*S. aureus*, 2338	*S. aureus*, ssc	*M. Globosa*, 42; *E. Coli*, 5; others, <5	11400
**108**	V2	*S. aureus*, 80	*S. aureus*, ssc	*Arthrobacter sp.*, 6; others, <6	2351
**108**	V3	S. aureus, 369	*S. aureus*, sparse	*M. Globosa*, 77; *Arthrobacter sp.*, 3; Others, <3	38980
**108**	V4	*S. aureus*, 4;	*S. aureus*, ssc	*E. Coli*, 25 *C. Acnes*, 4; others, <4	59
**108**	V5	*S. aureus*, 182	*S. aureus*, sparse	*E. Coli*, 33; *B. Subtilis*, 2; others,1	83
**108**	EN	Negative	NA	Negative	

**108**	NTC	Negative	NA	Negative	
**109**	VB	*S. aureus*, 70	*S. aureus*, ssc	*M. globosa*, 22; *E.coli*, 17; *C. acnes*, 7; *Arthrobacter sp.*, 4; *B. subtilis*, 2	16781
**109**	V2	*S. aureus*, 3	*S. aureus*, ssc	*M. globosa*, 27; *E. coli*, 22; B. subtilis, 10; Arthrobacter sp., 4	21171
**109**	V3	*S. aureus*, 2	*S. aureus*, ssc	*E. coli*, 16; *Arthrobacter sp.*, 5; *M. globosa*, 16; *C. acnes*, 3; *B. subtilis*, 3	25931
**109**	V4	*S. aureus*, 1	*S. aureus*, ssc	*E. coli*, 16; *Arthrobacter sp.*, 3; *B. subtilis*, 2	323
**109**	V5	Negative	*S. aureus*, ssc	*E. coli*, 9; *Arthrobacter sp.*, 2; *B. subtilis*, 3	56
**109**	EN	Negative	NA	*E coli*, 7; *B. subtilis*, 5	3
**109**	NTC	Negative	NA	Negative	5

**110**	VB	Negative	*S. aureus*, ssc	*M. globosa*, 2; *E. coli*, 2;<br>others, <2	38222
**110**	V2	Negative	*S. aureus*, ssc	*M. globosa*, 56; *E. coli*, 9; others, 1	1029
**110**	V3	Negative	*S. aureus*, ssc	*M. globosa*, 9; *E. coli*, 5; others, 1	2043
**110**	V4	Negative	*S. aureus*, ssc	*E. coli*, 8; others, 1	28
**110**	V7	Negative	*S. aureus*, ssc	*E. coli*,7; other, 1	1
**110**	NTC	Negative	NA	Negative	
**111**	VB	*S. aureus*, 537	*S. aureus*, rich	*M. globosa*, 12; others, <5	945
**111**	V2	*S. aureus*, 5683	*S. aureus*, rich	*M. globosa*, 141; *E. coli*, 5; others, <5	75837
**111**	V4	*S. aureus*, 217057;<br>*S. argenteus* 68;<br>*S. lugdunensis*, 53;<br>*S. epidermidis,* 42;<br>*S. pseudointermedius,* 30	*S. aureus*, rich	*E. coli*, 19; others <19	1799
**111**	V5	*S. aureus*, 55397;	*S. aureus*, rich	*M. globosa*, 24; *E. coli*, 16;	15138

		S. lugdunensis, 17		others <16	
**111**	V6	*S. aureus*, 2875	*S. aureus*, rich	*C. acnes*, 15; *E.coli*, 11;<br>*M. globosa*, 4; others, <4	2064
**111**	NTC	Negative	NA	*E. coli*, 1; *B. mycoides*, 1	
**112**	VB	*S. aureus*, 19	*S. aureus*, sparse	others, 1	29
**112**	V2	*S. aureus*, 6150	*S. aureus*, sparse	*M. globosa*, 15; *E. coli*, 8; others <3	8022
**112**	V3	*S. aureus*, 3326;<br>*S. lugdunensis*, 4	*S. aureus*, rich	others, <4	154
**112**	V4	*S. aureus*, 143	*S. aureus*, sparse	*M. globosa*, 9; *E. coli*, 4; others <4	2334
**112**	NTC	Negative	NA	Negative	
**114**	V2	*S. aureus*, 190119;<br>*S. argenteus*, 64;<br>*S. lugdunensis*, 27	*S. aureus*, mod	many others, <25	3524
**114**	V3	*S. aureus*, 582103;<br>*S. argenteus*, 113;<br>*S. lugdunensis*, 42;	*S. aureus*, rich	many others <25	449

		S. epidermidis, 29			
**114**	V4	*S. aureus*, 360674;<br>*S. argenteus*, 56;<br>*S. lugdunensis*, 18	*S. aureus*, mod	many others <25	70
**114**	V5	*S. aureus*, 52	*S. aureus*, rich	many others <3	5397
**114**	V6	*S. aureus*, 526710;<br>*S. argenteus*, 150;<br>*S. lugdunensis*, 68;<br>*S. epidermidis*, 39	*S. aureus*, mod	many others <30	142
**114**	EN	*S aureus*, 29	NA	many others <3	11
**114**	NTC	Negative	NA	*S. aureus*, 7; *M. oryzae*,1	
**115**	VB	*C. acnes*, 23	*C. acnes*, ssc, anaerobic	*E. coli*, 4; others 1	880
**115**	V2	*C. acnes*, 11	Culture negative	*S. equorum*, 3; others, <3	3722
**115**	V3	*C. acnes*, 31	Culture negative	*M. globosa*, 22; *E. coli*, 10;<br>*Klebsiella pneumoniae*, 4;<br>others, <4	9722

**115**	V4	*C. acnes*, 30	*C. acnes*, ssc, anaerobic	*E. coli*, 15; *Arthrobacter sp.*, 3;<br>others, <3	44
**115**	V5	*C. acnes*, 60	*C. acnes*, ssc, anaerobic	*E. coli*, 19; *M. globosa*, 3;<br>others, <3	1105
**115**	EN	Negative	NA	*E. coli*, 7;<br>*C. jeikeium*, 1	
**115**	NTC	Negative	NA	Negative	
**116**	VB	*S. aureus*, 18;<br>*S. epidermidis*, 4;	*S. aureus*, ssc	*E. coli*, 37;<br>*B. subtilis*, 6	684
**116**	V2	*S. aureus*, 13277;<br>*S. argenteus*, 15;<br>*S. nepalensis*, 12;<br>*S. epidermidis*, 11;<br>*S. agnetis*, 10;<br>*S. haemolyticus*, 6	*S. aureus*, sparse	Negative	34
**116**	V3	*S. aureus*, 10	*S. aureus*, ssc	*E. coli*, 13;	176

				M. osloensis, 3;<br>*B. subtilis*, 2	
**116**	V4	*S. aureus*, 1	*S. aureus*, ssc	*E. coli*, 24; *B. subtilis*, 5	875
**116**	R4	*S. aureus*, 450<br>*S. epidermidis*, 1	*S. aureus*, sparse	*E. coli*, 23; *B. subtilis*, 7	3226
**116**	EN	*S. epidermidis*, 1	NA	*E. coli*, 8; others, 1	1
**116**	NTC	Negative	NA	Negative	1
**117**	VB	*S. aureus*, 150	*S. aureus*, mod	Others, 2	124
**117**	V2	*S. aureus*, 8	*S. aureus*, sparse	Others, 2	1634
**117**	V3	*S. aureus*, 70;<br>*S. epidermidis*, 2	*S. aureus*, sparse	Others, 2	410
**117**	V4	*S. aureus*, 6	*S. aureus*, ssc	Others, 2	102
**117**	V5	*S. aureus*, 156;<br>*S. epidermidis*, 4	*S. aureus*, sparse	Negative	66
**117**	NTC	Negative	NA	Others, 1	20
**118**	VB	Negative	Culture negative	Negative	1821

**118**	V2	Negative	Culture negative	Negative	417
**118**	V3	Negative	Culture negative	Negative	1077
**118**	V4	Negative	Culture negative	*Ralstonia insidiosa*, 1	1835
**118**	V5	Negative	Culture negative	*Yersinia enterocolitica*, 1	8570
**118**	EN	Negative	Culture negative	IA	IA
**118**	NTC	Negative	NA	*E. coli*, 1	0
**120**	VB	Negative	*S. caprae*, ssc	*E. coli*, 85; *B. subtilis*, 18; *Arthrobacter sp.*, 13; *Mucilaginibacter sp.*, 6; *B. mycoides*, 4; others < 4	9660
**120**	V2	Negative	*S. caprae*, ssc	*E. coli*, 66; *B. subtilis*, 18; *Arthrobacter sp.*, 11; *Mucilaginibacter sp.*, 3; *B. mycoides*, 2; others,< 2	28
**120**	V3	Negative	*S. caprae*, ssc	*E. coli*, 66; *B. subtilis*, 18; *Arthrobacter sp.*, 11;	2848

				Mucilaginibacter sp., 3; *B. mycoides*, 2; others < 2	
**120**	V4	Negative	*S. caprae*, ssc	*E. coli*, 44; *B. subtilis*, 14; *Arthrobacter sp.*, 8; *Mucilaginibacter sp.*, 5; *B. mycoides*, 3	220
**120**	V5	Negative	*S. caprae*, ssc	*E. coli*, 57; *B. subtilis*, 18; *Arthrobacter sp.*, 5; *Mucilaginibacter sp.*, 6; *B. mycoides*, 4; others < 4	34
**120**	EN	Negative	NA	*E. coli*, 7; *Arthrobacter sp.*, 5; *C. acnes* 2; *B. subtilis* 1; *B. mycoides* 1, others, 1	4
**120**	NTC	Negative	NA		1
**121**	V2	Numerous GPAC;	*S. aureus*, ssc; *Peptoniphilus*	>2000 distinct species/subspecies, apparent	4998

		S. aureus, 984	*harei*, mod	2nd infection	
**121**	V3	Numerous GPAC; *S. aureus*, 1	*S. aureus*, broth; *Peptoniphilus harei*, sparse	>2000 distinct species/subspecies, apparent 2nd infection	144506
**121**	V4	Numerous GPAC	*S. aureus*, broth; *Peptoniphilus harei*, ssc	>2000 distinct species/subspecies, apparent 2nd infection	584
**121**	V6	Numerous GPAC	*S. aureus*, broth; *Peptoniphilus harei*, ssc	>2000 distinct species/subspecies, apparent 2nd infection	305
**121**	V8	Numerous GPAC; *S. aureus*, 1	Culture negative	>2000 distinct species/subspecies, apparent 2nd infection	27903
**121**	EN	Negative	NA	*E. coli*, 14; *S. mitis*, 5; *S. oralis*, 3; *B. subtilis*, 3; *C. acnes*, 3; others <3	67
**121**	NTC	Negative	NA	*E. coli*, 5; *B subtilis*, 1	5
**122**	VB	Negative	*S. aureus*, sparse	*M. globosa*, 181; others <1	10187
**122**	V2	*S. aureus*, 117	*S. aureus*, ssc	*S. aureus*, 117; *S. parasanguinis*,1	9

**122**	V3	*S. aureus*, 3480	*S. aureus*, rich	*M. globosa*, 154; *E. coli*, 9; *Mucilaginibacter sp.*, 5; others <5	31949
**122**	V4	*S. aureus*, 1269	*S. aureus*, ssc	E. coli, 4; others <4	1589
**122**	V5	*S. aureus*, 99	*S. aureus*, sparse	*M. globosa*, 27; *E. coli*, 5; others <5	2398
**122**	EN	Negative	NA	*E. coli*, 10; *M. osloensis*,3; others <3	9
**122**	NTC	Negative	NA	Negative	
**123**	VB	*S. aureus*, 15	*S. aureus*, ssc	*E. coli*, 10; *M. globosa*, 14; others, 1	56
**123**	V2	Negative	*S. aureus*, ssc; β-hemolytic streptococci, broth	*M. globosa*, 14; *E. coli*, 2; others, 1	6646
**123**	V3	*S. aureus*, 2	*S. aureus*, ssc; β-hemolytic streptococci, broth	*E. coli*, 6; others, 1	36
**123**	V4	*S. aureus*, 8	*S. aureus*, ssc	*E. coli*, 2; *S. oralis* 2; others, 1	70
**123**	V5	Negative	*S. aureus*, ssc	Negative	2

**123**	EN	Negative	NA	*E. coli*, 2; *C. acnes*, 2; others, 1	56
**123**	NTC	Negative	NA	Negative	0
**124**	VB	Negative	*S. epidermidis*, broth; *Corynebacterium amycolatum* and *Neisseria subflava*, broth (both presumed contamination)	*C. acnes*, 2; *E. coli*, 2	12
**124**	V2	Negative	Culture negative	*C. acnes*, 11	7
**124**	V3	Negative	Culture negative	*C. acnes*, 3;<br>*Propionibacterium sp.*, 2;<br>*C. granulosum*, 2	0
**124**	V4	Negative	*S. epidermidis*, broth	*C. acnes*, 6	33
**124**	V5	Negative	Culture negative	Negative	0
**124**	EN	Negative	NA	*C. acnes*, 2	10
**124**	NTC	Negative	NA	Negative	NA
**125**	VB	Negative	*S. aureus*, ssc	*E. coli*, 2; others, 1	1
**125**	V2	*S. aureus*, 2	Culture negative	*E. coli*, 6; *M. globosa*, 4; others, 1	222

**125**	V3	Negative	*M. luteus*, broth;<br>*S. aureus*, broth	*Arthrobacter sp.*, 1	12
**125**	V4	*S. aureus*, 1300	*M. luteus*, broth	*E. coli*, 5; others, <5	1943
**125**	EN	Negative	NA	*E. coli*, 4; others, 1	2
**125**	NTC	Negative	NA	Negative	0
**127**	VB	*S. aureus*, 4	*S. aureus*, sparse	others, 1	8
**127**	V2	*S. aureus*, 922;	*S. aureus*, rich	others, 2	310
**127**	V3	*S. aureus*, 2256	*S. aureus*, rich	others, 1	221
**127**	V4	*S. aureus*, 103	*S. aureus*, mod		17
**127**	V5	*S. aureus*, 734	*S. aureus*, rich	others, 1	30
**128**	VB	*S. aureus*, 9	*S. aureus*, sparse	others, 1	95
**128**	V2	*S. aureus*, 304	*S. aureus*, mod	others, 1	611
**128**	V3	*S. aureus*, 1	*S. aureus*, mod	*M. Osloensis*, 1	6
**128**	V4	*S. aureus*, 2	*S. aureus*, sparse	others, 1	19
**128**	V5	*S. aureus*, 31618	*S. aureus*, sparse	*S. Lugdunensis*, 17; others <17	2240
**128**	EN	Negative	NA	others, 1	1

**128**	NTC	Negative	NA	*S. Aureus*, 2	0
**130**	VB	*S. aureus*, 452	*S. aureus*, broth	*M. globosa*, 10; others 2 reads	656
**130**	V2	*S. aureus*, 12	*S. aureus*, broth	*M. globosa*, 18; *E. coli*, 4; others 1	1019
**130**	V3	*S. aureus*, 141	*S. aureus*, ssc	*A. oris*, 51; *V. parvula*, 43; *R. dentocariosa*, 34; *S. sanguinis*, 33; *M. globosa*, 7; *Cutibacterium sp.*, 31; *N. sicca*, 21; *F. nucleatum*, 9; *S. gordonii*, 5; others <5	8556
**130**	NTC	Negative	NA	Negative	11
**133**	VB	Negative	β-hemolytic streptococci, broth	*E. coli*, 9; *Klebsiella sp.*, 9; others <2	85
**133**	V2	*S. agalactiae*, 57	β-hemolytic streptococci, broth	*M. osloensis*, 11; *E. coli*, 5; others <5	275
**133**	V3	*S. agalactiae*, 131	β-hemolytic streptococci, rich	others, 1	53

**133**	V4	*S. agalactiae*, 30	β-hemolytic streptococci, sparse	*M. osloensis*, 5; others <5	199
**133**	V5	Negative	β-hemolytic streptococci, ssc	*E. coli*, 7; *F. nucleatum*, 6; others <5	37
**133**	EN	Negative	NA	*C. acnes*, 45; *S. capitis*, 7; others, <3	7
**133**	NTC	Negative	NA	*B. subtilis*, 1	0
**134**	V3	Negative	β-hemolytic streptococci, mod	*M. globosa*, 271; *Arthrobacter sp.*, 2; others <2	7556
**134**	V4	Negative	β-hemolytic streptococci, ssc	*E. coli*, 2; others <2	90
**134**	V5	Negative	β-hemolytic streptococci, sparse	*M. globosa*, 5; *E. coli*, 1	431
**134**	V6	*S. agalactiae*, 33	β-hemolytic streptococci, mod	*Arthrobacter sp.*, 2; others, <2	715
**134**	EN	Negative	NA	*E. coli*, 2; others <2	2
**134**	NTC	Negative	NA	Negative	0

**135**	VB	*S. dysgalactiae*, 17;<br>*S. agalactiae*, 3;<br>*S. mitis*, 3;<br>*S. pyogenes*,3	β-hemolytic streptococci, ssc	*M. globosa*, 68; *E. coli*, 18;<br>*M. osloensis*, 11; others,3	35999
**135**	V2	*S. dysgalactiae*, 240706,<br>*S. pyogenes*, 10761;<br>*S. agalactiae*, 8037;<br>*S. anginosis*, 2038;<br>*S. lutetiensis*, 410;<br>*S. gallolyticus*, 342;<br>*S. mutans*, 193;<br>*S. gordonii*, 188	β-hemolytic streptococci, ssc	*A. mediterraneensis*, 883;<br>*M. vaginalis*, 452;<br>others, <200	1159
**135**	V3	Negative	Culture negative	*E. coli*, 8; *M. globosa*, 3;<br>*B. subtilis*, 3; others,<3	631
**135**	V4	Negative	Culture negative	*M. globosa*, 338;	207550
**135**	V5	Negative	β-hemolytic streptococci,	*M. globosa*, 15; *E. coli*, 4;	7106

			ssc	*B. subtilis*, 3; others, <3	
**135**	EN	Negative	NA	*E. coli*, 30; *M. osloensis*, 11; *S. mitis*, 5; others, <5	475
**135**	NTC	Negative	NA	Negative	1
**136**	VB	Mitis group streptococci tot, 4; *S. capitis*, 3; *S. parasanguinis*, 2	*Granulicatella adiacens*, sparse	*M. globosa*, 68; *E. coli*, 41; *Arthrobacter sp.*, 4; others, <4	47624
**136**	V2	Mitis group streptococci tot, 18; staphylococci tot, 2	*Granulicatella adiacens*, sparse	*M. globosa*, 45; *E. coli*, 19, *B. subtilis*, 4; others, <4	29168
**136**	V3	Staphylococci tot, 1	*Granulicatella adiacens*, sparse; *Staphylococcus sp.*, broth	*E. coli*, 10; *M. globosa*, 7; *Arthrobacter sp.*, 5; others, <5	2351
**136**	V4	Mitis group streptococci tot, 4; staphylococci tot, 3	*Granulicatella adiacens*, ssc	*E. coli*, 21; *M. globosa*, 42; *Arthrobacter sp.*, 3; others, <3	24965
**136**	V5	Negative	*Granulicatella adiacens*, sparse	*E. coli*, 45; *M. globosa*, 244; *Arthrobacter sp.*, 3; *B. subtilis*, 3;	81413

				others, <3;	
**136**	EN	Negative	NA	*E. coli*, 4; *S. capitis*, 4; others, <4	27
**136**	NTC	Negative	NA	Negative	2
**137**	VB	Negative	Culture negative	Negative	15
**137**	V2	Negative	Culture negative	Negative	9
**137**	V3	Negative	Culture negative	Negative	2
**137**	V4	Negative	*Bacillus sp.,* broth (presumed contamination)	Negative	27
**137**	V5	Negative	Culture negative	Negative	8
**137**	EN	Negative	NA	Negative	1
**137**	NTC	Negative	NA	Negative	0
**139**	VB	*S. aureus*, 189	*S. aureus*, ssc	*E.coli*, 30; others <3	1277
**139**	V2	*S. aureus*, 12962; *S. argenteus*, 6	*S. aureus*, rich	*E. coli*, 19; others, <6	234
**139**	V3	*S. aureus*, 12423	*S. aureus*, rich	*E. coli*, 25; *C. acnes*, 7; others, <7	638

**139**	V4	*S. aureus*, 7362	*S. aureus*, sparse	*E. coli*, 18; *M. globosa*, 4;<br>others, < 4	3317
**139**	V5	*S. aureus*, 18	*S. aureus*, sparse	*E. coli*, 18; *C. acnes*, 5; others, <5	40
**139**	EN	Negative	NA	*E. coli*, 9; others, 1	14
**139**	NTC	Negative	NA	*B. subtilis*, 2; *E. coli*, 1;<br>*Arthrobacter sp.*, 1	1
**140**	VB	*S. aureus*, 714226;<br>*S. argenteus*, 122;<br>*S. epidermidis*, 108;<br>*S. lugdunensis*, 94;<br>*Staphylococcus sp.*, <50*	*S. aureus*, rich	*Bacillus sp.*, 12;<br>others, < 10	2785
**140**	VB	*S. aureus*, 661866;<br>*S. argenteus*, 142;<br>*S. epidermidis*, 57;<br>*S. lugdunensis*, 70;<br>*Staphylococcus sp.*, <50*	*S. aureus*, rich	*B. cerues.*,16;<br>*B. thuringiensis*, 14;<br>others, < 10	653

**140**	V2	*S. aureus*, 776139;<br>*S. argenteus*, 175;<br>*S. epidermidis*, 93;<br>*S. lugdunensis*, 106;<br>*Staphylococcus sp.*, <50*	*S. aureus*, rich	*B. cereus*, 38;<br>others, <10	372
**140**	V3	*S. aureus*, 18112;<br>*Staphylococcus sp.*, <50*	*S. aureus*, rich	*E. coli*, 3;<br>others, 1	382
**140**	V4	*S. aureus*, 18417;<br>*Staphylococcus sp.*, <50*	*S. aureus*, rich	*E. coli*, 4; *M. globosa*, 3;<br>others, <2	527
**140**	EN	Negative	NA	*S. aureus*, 2; *S. argenteus*, 5;<br>*E. coli*, 2; *C. acnes*,2; others, <2	1
**140**	NTC	Negative	NA	*S. aureus*, 2; *B. subtilis*, 2;<br>*E. coli*, 2; *Mucilaginibacter sp.*, 1	0
**141**	VB	*S. aureus*, 2123024;<br>*S. lugdunensis*, 542;<br>*S. epidermidis*, 431;	*S. aureus*, rich	*M. globosa*, 205;<br>*B. thuringiensis*,114;<br>*Bacillus sp.*, 72;	160982

		S. argenteus, 282;<br>*P. pseudointermedius*, 252;<br>*S. schleiferi*, 112;<br>*S. stepanovicii*, 100;<br>*S. simiae*, 99;<br>*S. capitis*, 73		*E. coli*, 52;<br>many others, <50	
**141**	V2	*S. aureus*, 1173	*S. aureus*, rich	*M. globosa*, 48; *E. coli*, 20;<br>*C. acnes*, 11; others, <10	32184
**141**	V3	*S. aureus*, 214625;<br>*S. lugdunensis*, 63	*S. aureus*, rich	many, < 50	1820
**141**	V4	*S. aureus*, 13941	*S. aureus*, rich	*M. globosa*, 58;<br>*E. coli*, 34; others, <10	23033
**141**	V5	*S. aureus*, 31057	*S. aureus*, rich	*E. coli*, 24; others< 20	432
**141**	EN	Negative	NA	*S. aureus*, 78; *E. coli*, 16;<br>*Arthrobacter sp.*, 5; others, <5	12
**141**	NTC	Negative	NA	*S. aureus*, 5; *E. coli*, 1;	0

				Alkalilimnicaola ehrlichii, 1	
**142**	VB	*S. epidermidis*, 8;<br>*S. aureus*, 7	Culture negative	*C. acnes*, 128; *E. coli*,61;<br>*M. globosa*, 28; *B. subtilis*, 17;<br>*Arthrobacter sp.*, 9;<br>*Mucilaginibacter sp.*, 4;<br>*S. capitis*, 3; *P. ananatis*, 2;<br>*M. osloensis*, 2; others <2	24252
**142**	V2	*S. epidermidis*, 3	Culture negative	*C. acnes*, 56; *E. coli*,57;<br>*M. globosa*, 4; *B. subtilis*, 10;<br>*Arthrobacter sp.*, 11;<br>*Mucilaginibacter sp.*, 4;<br>*B. mycoides*, 3; *S. mitis*, 3	959
**142**	V3	*S. oralis*, 4	Culture negative	*C. acnes*, 21; *E. coli*, 48;<br>*M. globosa*, 20; *B. subtilis*, 5;<br>*Arthrobacter sp.*, 3	10883
**142**	V4	Negative	Culture negative	*C. acnes*, 4; *E. coli*, 76;	518

				M. globosa, 20; *B. subtilis*, 25;<br>*Arthrobacter sp.*, 13;<br>*B. mycoides*, 4;<br>*Mucilaginibacter sp.*, 2	
**142**	V5	*S. oralis*, 4;	Culture negative	*C. acnes*, 6; *E. coli*,46;<br>*B. subtilis*, 19; Arthrobacter sp., 15;<br>*B. mycoides*, 3;<br>*Mucilaginibacter sp.*, 4	198
**142**	EN	Negative	NA	*C. acnes*, 8; *E. coli*, 22;<br>*B. subtilis*, 4; *Arthrobacter sp.*, 6;<br>*Mucilaginibacter sp.*, 2;<br>*B. mycoides*, 2	0
**142**	NTC	Negative	NA		0
**200**	VB	Negative control	Culture negative	*E. coli*, 37; *Arthrobacter sp.*, 13;<br>*M. osloensis*, 13; *B. subtilis*, 12;	843

				C. acnes, 9 *M. globosa*, 7; *Mucilaginibacter sp.*, 3	
**200**	V2	Negative control	Culture negative	*E. coli*, 68; *M. globosa*, 34; *Arthrobacter sp.*, 17; *B. subtilis*, 12; *Mucilaginibacter sp.*, 7; *M. osloensis, 5*; *C. acnes*, 5; *B. mycoides*, 3; *Pantoa ananatis*, 3; *S. gordonii*, 3	35266
**200**	V3	Negative control	Culture negative	*E. coli*, 40; *Arthrobacter sp.*, 7; *M. osloensis*, 3; *B. subtilis*, 4; *C. acnes*, 3; *M. globosa*, 32;	14882
**200**	V4	Negative control	Culture negative	*E. coli*, 37; *Arthrobacter sp.*, 6; *B. subtilis*, 13; *Mucilaginibacter sp.*, 7; *M. osloensis*, 5; *B. mycoides*, 3;	

**200**	V5	Negative control	Culture negative	*E.coli*, 29; *Arthrobacter sp.*, 20; *M. osloensis*, 4; *B. subtilis*, 10; *C. acnes*, 6; *M. globosa*, 14; *Mucilaginibacter sp.*, 4	10020
**200**	EN	Negative control	NA	Negative	
**200**	NTC	Negative control	NA	*E. coli*, 5;	5
**201**	VB	Negative control	Culture negative	others, <3	254
**201**	V2	Negative control	Culture negative	*M. globosa*, 3	450
**201**	V3	Negative control	Culture negative	others, <3	141
**201**	V4	Negative control	Culture negative	others, <3	97
**201**	V5	Negative control	Culture negative	others, <3	41
**201**	EN	Negative control	NA	others, <3	1
**201**	NTC	Negative control	NA	Negative	0
**202**	VB	Negative control	Culture negative	*E. coli*, 3; *M. globosa*, 4;	1783

**202**	V2	Negative control	Culture negative	*M. globosa*, 7;	510
**202**	V3	Negative control	Culture negative	*E. coli*, 12; *M. globosa*, 21;	12678
**202**	V4	Negative control	Culture negative	*E. coli*, 8	217
**202**	V5	Negative control	Culture negative	*E. coli*, 13; *M. globosa*, 40;	25376
**202**	EN	Negative control	NA	*E. coli*, 6	4
**202**	NTC	Negative control	NA	Negative	0
**203**	VB	Negative control	Culture negative	Negative	5
**203**	V2	Negative control	Culture negative	Negative	6
**203**	V3	Negative control	Culture negative	*M. globosa*, 318;	5599
**203**	V4	Negative control	Culture negative	*M. osloensis*, 3	3886
**203**	V5	Negative control	Culture negative	*M. globosa*, 23; *E. coli*, 3	1891
**203**	EN	Negative control	NA	Negative	0
**203**	NTC	Negative control	NA	Negative	0
**204**	VB	Negative control	Culture negative	*E. coli*, 7; *M. globosa*, 75; *Arthrobacter sp.*, 2;	32185

				K. pneumoniae, 1; *M. osloensis*, 11	
**204**	V2	Negative control	Culture negative	*E. coli*, 15; *M. globosa*, 16; *Arthrobacter sp.*, 1; *C. acnes*, 3; *S. capitis*, 3; *Klebsiella pneumoniae* 3 *M. osloensis*, 17; *Acinetobacter baumanii*, 2	20967
**204**	V3	Negative control	Culture negative	*E. coli*, 2; *M. globosa*, 727; *Arthrobacter sp.*, 2	13571
**204**	V4	Negative control	Culture negative	*E. coli*, 4; *M. globosa*, 132; *Arthrobacter sp.*, 2	6102
**204**	V5	Negative control	Culture negative	*E. coli*, 4; *B. subtilis*, 2; *Mucilaginibacter sp.*, 2	210
**204**	EN	Negative control	NA	*M. osloensis*, 10; *E. coli*, 3	14
**204**	NTC	Negative control	NA	*E. coli*,1; *B. subtilis*, 1	0

**S. pseudointermedius, S. schleiferi, S. capitis, S. stepanovicii, S. simiae, S. pasteuri, S. simulans, S. lutrae, S. saprophyticus, S. nepalensis, S. piscifermentans, S. haemolyticus, S. pettenkoferi, S. sciuri, S. xylosus, S. argenteus*

Table S2. Bioinformatic program versions used.

Program	Version
MinKNOW	1.15.4 to v. 3.6.5
Guppy	3.0.3 to v. 3.2.10

EPI2ME	2.57.1769546 to v. 2019.7.9.2549693

Table S3. Nanopore sequencing QC metrics, totals for each patient.

Patient ID	Total reads	Total bases	Mean quality score	Mean read length (bp)
**101**	4567	14.1 Mb	10.7	3084
**104**	52607	144.1 Mb	12.84	2739
**105**	84234	263.3 Mb	12.1	3126
**107**	9404	17.5 Mb	9.59	1863
**108**	60125	141.6 Mb	12.51	2354
**109**	385891	1.3 Gb	12.74	3296
**110**	44309	124.6 Mb	12.54	2811
**111**	394935	1.1 Gb	12.46	2779
**112**	21750	60.9 Mb	12.48	2800
**114**	44309	124.6 Mb	12.54	2811

**115**	17165	41.7 Mb	12.17	2430
**116**	22074	61.4 Mb	11.84	2780
**117**	1463935	3.8 Gb	8.07	2621
**118**	73076	89.3 Mb	5.66	1222
**120**	81223	345.9 Mb	11.77	4258
**121**	458946	1.9 Gb	12.8	4186
**122**	56248	122.2 Mb	11.56	2172
**123**	7750	20.5 Mb	11.84	2647
**124**	758	560.9 Kb	8.12	739
**125**	4423	10.8 Mb	11.86	2445
**127**	71026	235.4 Mb	10.31	3313
**128**	37382	111.4 Mb	12.38	2980
**130**	3880728	14.5 Gb	10.53	3738
**133**	1536	3.2 Mb	11.46	2091
**134**	10624	22.4 Mb	11.63	2107
**135**	535666	2 Gb	12.41	3686

**136**	195174	604.9 Mb	12.53	3099
**137**	13690	14.6 Mb	4.46	1069
**139**	41736	139.9 Mb	12.54	3351
**140**	2273296	9.8 Gb	12.87	4324
**141**	2744512	11.9 Gb	12.89	4335
**142**	30010	103.3 Mb	12.44	3441
**200**	67637	162.5 Mb	12.59	2402
**201**	2288	2.1 Mb	9.99	931
**202**	43111	97.8 Mb	12.39	2267
**203**	15396	30.8 Mb	11.42	2002
**204**	79478	198.7 Mb	12.26	2499

Table S4. Overview of all patients whose biopsies were positive for either an antimicrobial resistance (AMR) phenotype from conventional antibiotic susceptibility testing (AST) or AMR genotype from shotgun metagenomic sequencing. Definitions used to categorize the isolates' AST results were (S) sensitive, intermediate (I) or resistant (R). Identity cut-off for AMR genotype match was set at 90%. *Other* indicates detected AMR genes not reflected in AST phenotype, or putative AMR genes of uncertain function. *None detected* indicates that patient's biopsies were sequencing positive, but no AMR genes were detected. *Sequencing negative* indicates that sequencing failed to detect the cultured pathogen, and hence its AMR gene(s).

ID	AST (phenotype)	AMR Genotype (reads)
**104**	**Find 1:** ***S. lugdunensis***	None detected
	Ciprofloxacin (S)	
	Erythromycin (S)	
	Fusidic acid (S)	
	Gentamicin (S)	
	Clindamycin (S)	
	Chloramphenicol (S)	
	Linezolid (S)	
	Oxacillin (S)	
	Rifampicin (S)	
	Penicillin (R)	
	Tetracycline (S)	
	Trimethoprim/sulfamethoxazole (S)	
	**Find 2:** ***C. acnes***	

	Clindamycin (S)	
	Chloramphenicol (S)	
	MeroPenicillin em (S)	
	Metronidazole (R)	
	Penicillin	
**105**	**Find 1:** ***S. aureus***	Sequencing negative
	Ciprofloxacin (S)	
	Erythromycin (S)	
	Fusidic acid (R)	
	Gentamicin (S)	
	Clindamycin (S)	
	Chloramphenicol (S)	
	Linezolid (S)	
	Oxacillin (S)	
	Rifampicin (S)	
	Penicillin (R)	
	Teicoplanin (S)	
	Tetracycline (S)	
	Trimethoprim/sulfamethoxazole (S)	
	Vancomycin (S)	
	**Find 2:** ***S. epidermidis***	
	Ciprofloxacin(S)	
	Erythromycin (S)	

	Fusidic acid (R)	
	Gentamicin (S)	
	Clindamycin (S)	
	Chloramphenicol (S)	
	Linezolid (S)	
	Oxacillin (S)	
	Rifampicin (S)	
	Teicoplanin (S)	
	Tetracycline (S)	
	Trimethoprim/sulfamethoxazole (S)	
	Vancomycin (S)	
**107**	**Find:** ***S. epidermidis***	Sequencing negative
	Ciprofloxacin (R)	
	Erythromycin (R)	
	Fusidic acid (R)	
	Gentamicin (S)	
	Clindamycin (R)	
	Chloramphenicol (S)	
	Linezolid (S)	
	Oxacillin (R)	
	Rifampicin (S)	
	Teicoplanin (S)	
	Tetracycline (S)	

	Trimethoprim/sulfamethoxazole (R)	
	Vancomycin (S)	
108	**Find:** ***S. aureus***	
	Ciprofloxacin(S)	
	Erythromycin (S)	
	Fusidic acid (S)	
	Gentamicin (S)	
	Clindamycin (S)	
	Chloramphenicol (S)	
	Linezolid (S)	
	Oxacillin (S)	
	Rifampicin (S)	
	Penicillin (R)	*blaZ* (26)
	Tetracycline (S)	
	Trimethoprim/sulfamethoxazole (S)	
	other	*sav1866* (7)
109	**Find:** ***S. aureus***	None detected
	Ciprofloxacin (S)	
	Erythromycin (S)	
	Fusidic acid (S)	
	Gentamicin (S)	
	Clindamycin (S)	
	Chloramphenicol (S)	

	Linezolid (S)	
	Oxacillin (S)	
	Rifampicin (S)	
	Penicillin (R)	
	Tetracycline (S)	
	Trimethoprim/sulfamethoxazole (S)	
**111**	**Find:** ***S. aureus*** (S)	
	Ciprofloxacin (S)	
	Erythromycin (S)	
	Fusidic acid (S)	
	Gentamicin (S)	
	Clindamycin (S)	
	Chloramphenicol (S)	
	Linezolid (S)	
	Oxacillin (S)	
	Rifampicin (S)	
	Penicillin (R)	*blaZ* (3390)
	Tetracycline (S)	
	Trimethoprim/sulfamethoxazole (S)	
	other	*tet38* (480), *sav1866* (394), *mepA* (376), *mgrA* (296), *arlS* (281), *mepR* (94)
**112**	**Find:** ***S. aureus***	
	Ciprofloxacin (S)	

	Erythromycin (S)	
	Fusidic acid (S)	
	Gentamicin (S)	
	Clindamycin (S)	
	Linezolid (S)	
	Oxacillin (S)	
	Rifampicin (S)	
	Penicillin (R)	
	Tetracycline (S)	
	Trimethoprim/sulfamethoxazole (S)	
	other	*sav1866* (15), *tet38* (15), *mgrA* (10), *arlS* (7), *mepA* (5), *mepR* (5)
**114**	**Find:** ***S. aureus***	
	Ciprofloxacin (S)	
	Erythromycin (S)	
	Fusidic acid (S)	
	Gentamicin (S)	
	Clindamycin (S)	
	Linezolid (S)	
	Oxacillin (S)	
	Rifampicin (S)	
	Penicillin (R)	*blaZ* (943)
	Tetracycline (S)	

	Trimethoprim/sulfamethoxazole (S)	
	Other	*blaZ* (943) *tet38* (2403), *sav1866* (1622), *mgrA* (1257), *arlS* (1154), *arlR* (344), *mepR* (338)
**116**	**Find:** ***S. aureus***	
	Ciprofloxacin(S)	
	Erythromycin (S)	
	Fusidic acid (S)	
	Gentamicin (S)	
	Clindamycin (S)	
	Linezolid (S)	
	Oxacillin (S)	
	Rifampicin (S)	
	Penicillin (R)	*blaZ (156), bla*$_{TEM-4}$ (55)
	Tetracycline (S)	
	Trimethoprim/sulfamethoxazole (S)	
	Other	*sav1866* (17), *tetC* (813) *bla*$_{TEM-4}$ (55), *mepA* (10), *arlS* (19), *mgrA* (8)
**117**	**Find:** ***S. aureus***	
	Ciprofloxacin (R)	*mgrA*, 1 (87% match)
	Erythromycin (S)	
	Fusidic acid (S)	
	Gentamicin (S)	

	Clindamycin (S)	
	Linezolid (S)	
	Oxacillin (S)	
	Rifampicin (S)	
	Penicillin (R)	
	Tetracycline (S)	
	Trimethoprim/sulfamethoxazole (S)	
**120**	**Find:** ***S. caprae***	Sequencing negative
	Ciprofloxacin (S)	
	Erythromycin (S)	
	Fusidic acid (R)	
	Gentamicin (S)	
	Clindamycin (S)	
	Linezolid (S)	
	Oxacillin (S)	
	Rifampicin (S)	
	Teicoplanin (S)	
	Tetracycline (S)	
	Trimethoprim/sulfamethoxazole (S)	
	Vancomycin (S)	
**121**	**Find:** ***S. aureus***	None detected
	Ciprofloxacin (S)	
	Erythromycin (S)	

	Fusidic acid (S)	
	Gentamicin (S)	
	Clindamycin (S)	
	Linezolid (S)	
	Oxacillin (S)	
	Rifampicin (S)	
	Penicillin (R)	
	Tetracycline (S)	
	Trimethoprim/sulfamethoxazole (S)	
**122**	**Find:** ***S. aureus***	
	Ciprofloxacin (S)	
	Erythromycin (S)	
	Fusidic acid (S)	
	Gentamicin (S)	
	Clindamycin (S)	
	Linezolid (S)	
	Oxacillin (S)	
	Rifampicin (S)	
	Penicillin (R)	*blaZ* (8)
	Tetracycline (S)	
	Trimethoprim/sulfamethoxazole (S)	
	other	*tet38* (12), *sav1866* (9), *mgrA* (6)
**123**	**Find 1:** ***S. aureus***	None detected

	Ciprofloxacin (S)	
	Erythromycin (S)	
	Fusidic acid (S)	
	Gentamicin (S)	
	Clindamycin (S)	
	Linezolid (S)	
	Oxacillin (S)	
	Rifampicin (S)	
	Penicillin (R)	
	Tetracycline (S)	
	Trimethoprim/sulfamethoxazole (S)	
	**Find 2: β-hemolytic streptococci**	
	Erythromycin (S)	
	Clindamycin (S)	
	Oxacillin (S)	
	Penicillin (S)	
	Trimethoprim/sulfamethoxazole (S)	
	Vancomycin (S)	
**124**	**Find:** ***S. epidermidis***	Sequencing negative
	Ciprofloxacin (R)	
	Erythromycin (R)	
	Fusidic acid (S)	
	Gentamicin (R)	

	Clindamycin (R)	
	Linezolid (S)	
	Oxacillin (R)	
	Rifampicin (S)	
	Teicoplanin (S)	
	Tetracycline (S)	
	Trimethoprim/sulfamethoxazole (R)	
	Vancomycin (S)	
**125**	**Find:** ***S. aureus***	
	Ciprofloxacin (S)	
	Erythromycin (S)	
	Fusidic acid (S)	
	Gentamicin (S)	
	Clindamycin (S)	
	Linezolid (S)	
	Oxacillin (S)	
	Rifampicin (S)	
	Penicillin (R)	*blaZ* (2)
	Tetracycline (S)	
	Trimethoprim/sulfamethoxazole (S)	
	other	*mepA* (5), *sav1866* (3), *tet38* (2)
**127**	**Find:** ***S. aureus***	
	Ciprofloxacin (R)	*arlS* (11), *mgrA* (6),

	Erythromycin (S)	
	Fusidic acid (S)	
	Gentamicin (S)	
	Clindamycin (S)	
	Linezolid (S)	
	Oxacillin (S)	
	Rifampicin (S)	
	Penicillin (S)	
	Tetracycline (S)	
	Trimethoprim/sulfamethoxazole (S)	
	other	*sav1866* (8)
**128**	**Find:** ***S. aureus***	
	Ciprofloxacin (I)	*mgrA* (34), *arlS* (40), *arlR* (9)
	Erythromycin (S)	
	Fusidic acid (S)	
	Gentamicin (S)	
	Clindamycin (S)	
	Linezolid (S)	
	Oxacillin (S)	
	Rifampicin (S)	
	Penicillin (R)	*blaZ* (28)

	Tetracycline (R)	*tetK* (28), *tet38* (50), *sav1866* (52), *mepR* (12), *mepA* (36), *mgrA* (34)
	Trimethoprim/sulfamethoxazole (S)	
**130**	**Find:** ***S. aureus***	
	Ciprofloxacin (S)	
	Erythromycin (S)	
	Fusidic acid (S)	
	Gentamicin (S)	
	Clindamycin (S)	
	Linezolid (S)	
	Oxacillin (S)	
	Rifampicin (S)	
	Penicillin (R)	
	Tetracycline (S)	
	Trimethoprim/sulfamethoxazole (S)	
	other	*mepA* (2)
**135**	**Find: B-hemolytic streptococci**	
	Erythromycin (R) , Clindamycin (R)	*lmrP*, 620 (88.8% match)
	Penicillin (S)	
**139**	**Find:** ***S. aureus***	
	Ciprofloxacin (S)	
	Erythromycin (S)	
	Fusidic acid (S)	

	Gentamicin (S)	
	Clindamycin (S)	
	Linezolid (S)	
	Oxacillin (S)	
	Rifampicin (S)	
	Penicillin (R)	*blaZ* (45)
	Tetracycline (S)	
	Trimethoprim/sulfamethoxazole (S)	
	other	*tet38* (95), *mepA* (56), *sav1866* (53), *arlS* (36), *mepR* (9), *arlR* (7)
**140**	**Find:** ***S. aureus***	
	Ciprofloxacin (S)	
	Erythromycin (S)	
	Fusidic acid (S)	
	Gentamicin (S)	
	Clindamycin (S)	
	Linezolid (S)	
	Oxacillin (S)	
	Rifampicin (S)	
	Penicillin (R)	*blaZ* (3392)
	Tetracycline (S)	
	Trimethoprim/sulfamethoxazole (S)	

	other	*tet38* (5251); *sav1866* (4451); *mepA* (4397); *mgrA* (3580); *arlS* (3521); *arlR* (856); *mepR* (773)
**141**	**Find:** ***S. aureus***	
	Ciprofloxacin (S)	
	Erythromycin (S)	
	Fusidic acid (S)	
	Gentamicin (S)	
	Clindamycin (S)	
	Linezolid (S)	
	Oxacillin (S)	
	Rifampicin (S)	
	Penicillin (R)	*blaZ* (3172)
	Tetracycline (S)	
	Trimethoprim/sulfamethoxazole (S)	
	other	*tet38* (6479), *sav1866* (4362), *mepA* (5456), *mgrA* (3431), *arlS* (3106), , *arlR* (754), *mepR* (1010)
